# *ADAR1* expression in different cancer cell lines and its change under heat shock

**DOI:** 10.1007/s13353-024-00926-4

**Published:** 2024-12-06

**Authors:** Dominika Adamczak, Michał Fornalik, Anna Małkiewicz, Julia Pestka, Andrzej Pławski, Paweł Piotr Jagodziński, Bartosz Kazimierz Słowikowski

**Affiliations:** 1 Department of Biochemistry and Molecular Biology, Poznan University of Medical Sciences, Święcickiego 6 Street, 60-781 Poznań, Poland; 2 Institute of Human Genetics, Polish Academy of Sciences, Strzeszyńska 32 Street, 60-479 Poznań, Poland

**Keywords:** ADAR1, Stress response, Cancer, RNA editing

## Abstract

Adenosine deaminase acting on RNA 1 (ADAR1) plays an essential role in the development of malignancies by modifying the expression of different oncogenes. ADAR1 presents three distinct activities: adenosine-to-inosine RNA editing, modulating IFN pathways, and response to cellular stress factors. Following stressors such as heat shock, ADAR1p110 isoform relocates from the nucleus to the cytoplasm, where it suppresses RNA degradation which leads to the arrest of apoptosis and cell survival. In this study, we assessed the expression of ADAR1 across different cancer cell lines. We revealed that the presence of ADAR1 varies between cells of different origins and that a high transcript level does not reflect protein abundance. Additionally, we subjected cells to a heat shock in order to evaluate how cellular stress factors affect the expression of ADAR1. Our results indicate that ADAR1 transcript and protein levels are relatively stable and do not change under heat shock in examined cell lines. This research lays a groundwork for future directions on ADAR1-related studies suggesting in which types of cancer ADAR1 may be a promising target for novel therapeutic approaches.

## Introduction

Since their discovery in 1988 in *Xenopus* eggs (Bass and Weintraub 1988; Wagner et al. 1989), proteins from the ADAR (adenosine deaminase acting on RNA) family have been a subject of interest for many scientific teams. However, it would be an overstatement that we know everything about them, as new features are continuously being discovered.

In humans, three proteins from the ADAR family have been identified: ADAR1, ADAR2, and ADAR3, from which 1 and 2 have been found to exhibit catalytic activity (Cho et al. 2017). Our study was focused on ADAR1 as it is expressed in all mammalian cells (Kim et al. 1994). This protein is also crucial for embryonic development. *Adar1-/-* mice embryos die between embryonic days 11.5–12.5 and present a delay in development accompanied by abnormalities in the liver structure and impaired early liver hematopoiesis (Hartner et al. 2004). Two isoforms of this protein have been identified so far: ADAR1p110 and p150. The shorter p110 isoform is constitutively expressed in the nucleus, while the p150 is an interferon (IFN) inducible protein present in the nucleus and cytoplasm (Cho et al. 2017).

Despite this difference, they perform the same reaction: deamination of adenosine (A) at the C6 position, which converts it to inosine (Ins) (Bass and Weintraub 1988). This results in reading Ins as guanine during translation (Nishikura 2016; Porath et al. 2014), which may alter the newly synthesized protein’s function or the RNA molecule’s properties. A-to-I editing is the most common RNA modification in mammals (Bazak et al. 2014; Porath et al. 2014).

ADAR1 can target all dsRNA molecules longer than approximately 20 bp (Nishikura et al. 1991). However, studies have shown that it primarily acts on specific RNA molecules transcribed from *Alu* repeats (Athanasiadis et al. 2004), the most common short interspersed nuclear elements (SINEs) in our genome. Humans have about a million copies of them, making up around 10% of our genetic information (Cho et al. 2017). These sequences hybridize with other opposing *Alu* elements producing stable dsRNA regions, creating a perfect target for ADAR1. Most edited regions (up to 93%) lay in introns or non-coding regions (Porath et al. 2014). Nevertheless, studies focused on sequencing and screening for edited sites proved that similar editing can also occur in the coding regions of multiple genes, which sets the ground for further research on the effects of those modifications (Li et al. 2009; Peng et al. 2012; Porath et al. 2014; Wu et al. 2023).

Recently, several studies discovered more functions of ADAR, including RNA editing in viral infection and the response to cellular stress factors. In vitro experiments have shown that RNA modification inhibits the IFN-mediated apoptosis pathway and upregulates the transcription of multiple genes, which are IFN-stimulated (Liddicoat et al. 2015). Moreover, one investigation assessed the role of ADAR1 protein in apoptosis under cellular stress factors such as UV light and heat shock (Sakurai et al. 2017). Stress conditions such as UV radiation or heat shock cause the ADAR1 p110 to relocate from the nucleus to the cytoplasm, where it binds to dsRNA. However, its role in this context is likely unrelated to RNA editing but to inhibit Staufen-1 mediated mRNA degradation. This function is essential for cell survival, as the ADAR1-null cells die under these conditions. Moreover, ADAR1 regulates the expression of many genes, including those involved in cell cycle regulation, DNA repair, or apoptosis through this activity (Sakurai et al. 2017).

Another ADAR1 activity that indirectly contributes to cancer is interfering with apoptosis pathways induced by IFN. Ishizuka et al. showed significant inhibition of viability and increased apoptosis in *ADAR1* deficient cells after IFNβ and IFNγ stimulation (Ishizuka et al. 2019). This phenomenon may occur during viral infection when RNA molecules of a double-stranded structure containing A but not I appear in cells. These fragments are recognized by RNA sensing proteins, including protein kinase R (PKR) or melanoma differentiation-associated protein 5 (MDA5), which leads to inhibition of translation or induction of apoptosis (Gélinas et al. 2011; Liddicoat et al. 2015). In contrast, to prevent sensing self-RNA as exogenous, it undergoes modifications like A-to-I editing by ADAR1, which impairs the ability of these proteins to bind and eliminate dsRNA (Liddicoat et al. 2015). This mechanism mitigates excessive IFN outbursts and maintains homeostatic conditions inside the cells (Liddicoat et al. 2015).

The depletion of ADAR1 activity enhances the response to IFN and upregulates IFN-stimulated genes (ISG) during viral infection and IFN treatment (Liddicoat et al. 2015). Interestingly, ADAR1 itself is an IFN-inducible protein. Its activation results in lowering the excessive production of IFN, which may act as a self-limiting mechanism (Karki et al. 2021; Maas et al. 2006).

This inhibition of IFN protects cells against autoimmune diseases (i.e., Aicardi-Goutières disease, dyschromatosis symmetrica hereditaria (Wang et al. 2019), and psoriasis (Rice et al. 2012)) but hinders cancer recognition by the immune system (Rice et al. 2012) making ADAR1 a notable determinant of immunological checkpoint-blockade treatment resistance (Ishizuka et al. 2019).

Loss of *ADAR1* overcomes tumor resistance to PD-1 checkpoint blockade treatment, increasing therapy efficiency and survival rate in mice. Additionally, ADAR1 seems to be involved in response to radiotherapy and certain chemotherapeutic agents such as oxaliplatin and bleomycin, which stimulate an IFN-dependent pathway and upregulate the production of IFN and ISG, validating their effectiveness in the treatment (Miar et al. 2020; Minn 2015; Nemlich et al. 2013). *ADAR1* null cells after radiation and imiquimod, an immune response modifier, produced significantly more IFNβ, which led to noticeably lower viability (Ishizuka et al. 2019).

ADAR1 is a promising target for oncogenic treatment as silencing *ADAR1* decreases the aggressiveness of tumors (mainly characterized by self-renewal, proliferation, and migration), including thyroid cancer (Ramírez-Moya et al. 2021, 2020), glioblastoma (Jiang et al. 2022), and breast cancer (Kung et al. 2021; Liu et al. 2019). Promising results have been obtained for triple-negative breast cancer (TNBC), which lacks all molecular markers for targeted therapy and relies primarily on chemotherapy as a therapeutic option (Almansour 2022; Waks and Winer 2019). Furthermore, chemoresistance and *ADAR1* upregulation are associated. Liu et al. state that ADAR1 activity, among others, enhances *BRCA2* expression, which in turn promotes cis-platin resistance in intrahepatic cholangiocarcinoma. In addition, a notable reduction in tumor volume was observed when *ADAR1* knockdown and cisplatin treatment were combined (Liu et al. 2024). Similarly, depletion of *DDX-1* and therefore ADAR1 resulted in increased sensitivity of non-small lung cancer cells to cisplatin both in vitro and in vivo settings (Yang et al. 2023). In gastric cancer, resistance to standard treatments like 5-fluorouracil and cisplatin has been linked to ADAR1-mediated RNA A-to-I modifications. This resistance mechanism appears to arise from an increase in ADAR1 protein levels, which is suggested to result from the activation of the interferon/JAK2/STAT3 signaling pathway (Wong et al. 2023). It would be beneficial to assess whether *ADAR1* expression correlates with the level of inflammation or the level of IFN response to further develop new strategies involving this protein.

The RNA-editing activity of ADAR1 also contributes to cancer. A high level of A-to-I RNA editing correlates with high cancer grade and poor patient survival, as observed in patients with glioblastoma (Han et al. 2015), breast (Fumagalli et al. 2015; Nakano et al. 2017; Sagredo et al. 2018), thyroid (Ramírez-Moya et al. 2021), and kidney cancer (Wu et al. 2023).

For instance, common editing in the Ras homolog family member Q (RHOQ) promotes the invasion of colorectal cancer (Han et al. 2014). Similar results were obtained for hepatocellular carcinoma where the degree of antizyme inhibitor 1 (AZIN1) was related to carcinogenesis, initiation, and development (Chen et al. 2013). Another example of a hyper-edited transcript can be a tumor suppressor gene, bladder cancer-associated protein (BLCAP), which promotes cell proliferation in cervical cancer and hepatocellular carcinoma (Fritzell et al. 2018). In addition, RNA editing can also contribute to carcinogenesis by protecting certain transcripts from microRNA silencing, such as the human dihydrofolate reductase (Nakano et al. 2017).

However, in metastatic melanoma cells, the low A-to-I RNA editing promotes tumor progression via growth and metastasis (Nemlich et al. 2013; Shoshan et al. 2015). These potentially conflicting results indicate that more studies are required to understand this relationship.

Despite these findings, to our best knowledge, studies have yet to compare the level of ADAR1 across multiple cell lines and evaluate the effect of different cellular stress factors. Therefore, we hypothesized that the *ADAR1* expression differs in each cell line and changes depending on the specific stress factor, and we conducted a study to test this hypothesis.

## Materials and methods

### Cell culture

During this study, we utilized eight different cell lines that were provided by the Department of Biochemistry and Molecular Biology at Poznan University of Medical Sciences. These cell lines included lung cancer (A-549, Calu-1), cervical carcinoma (SiHa, HeLa), breast adenocarcinoma (MCF-7), colon carcinoma (HCT-116), and gastric carcinoma (AGS, HGC-27) lines. The cells were maintained in RPMI 1640/DMEM F12 (Sigma-Aldrich, St. Louis, MO) supplemented with 10% FBS and 1% antimycotic-antibiotic solution (Sigma-Aldrich, St. Louis, MO). In the early phase of the experiment, every cell line was grown on culture flasks. The cells were then collected and counted using EVE™ Automated Cell Counter (NanoEnTek, Seoul, South Korea) and trypan blue (Thermo Fisher, Waltham, USA) before being seeded into the appropriate culture vessels. The cultures were grown for 24 h under standard conditions: 37 °C and 5% CO_2_. To investigate the heat shock influence, cells were subjected to a temperature of 43 °C for 2 h. Then, cells were cultured in a standard way. Each experiment was performed in three biological replicates and included a control sample that was not subjected to heat shock treatment.

### Isolation of RNA, reverse transcription, and RT-qPCR

Total cellular RNA was extracted by using TRIzol® reagent (Thermo Fisher, Waltham, USA) according to the manufacturer’s protocol. The quantity and purity of the extracted RNA samples were determined spectrophotometrically using NanoDrop™ One (Thermo Fisher, Waltham, USA). Additionally, the quality and integrity of isolated RNA were assessed through agarose gel electrophoresis.

For cDNA synthesis, the reverse transcription was performed using MMLV reverse transcriptase (Thermo Fisher Scientific, Waltham, USA) with a mixture of oligo dT primers and random hexamers. Real-time quantitative polymerase chain reaction (RT-qPCR) analysis was carried out using SYBR Green I Master and Light Cycler®480 Real-Time PCR System (Roche Diagnostics GmbH, Mannheim, Germany).

To determine the efficiency of the reactions, standard curves were generated from a serial dilution of the cDNA template mix from all of the samples. Negative controls, including no-template, non-transcribed RNA, and genomic DNA, were utilized in every analysis. For calibration, 1 µl of cDNA template mix was used. Porphobilinogen deaminase *(PBGD*), RNA polymerase II subunit A (*POLR2A*), and human mitochondrial ribosomal protein L19 (*hMRPL19*) were used as the reference genes. Their expression geometric mean was utilized to standardize the mRNA level of *ADAR1.* The specificity of the SYBR Green PCR products was confirmed through melting curve analysis and agarose gel electrophoresis.

### Protein isolation and western blot

For the purpose of protein isolation, RIPA lysis buffer (Sigma-Aldrich, St. Louis, USA) enriched with cOmplete™ protease inhibitor cocktail (Roche Diagnostics GmbH, Mannheim, Germany) was used. Cells were collected and incubated with buffer for 30 min, followed by centrifugation at 10 000 × g for 10 min at 4 °C to remove cellular debris. Next, 30 µg of total protein was resuspended in sample loading buffer, incubated at 99 °C for 10 min, cooled on ice, and separated on 12% Tris–Glycine gels, using sodium dodecyl sulfate–polyacrylamide gel electrophoresis (SDS-PAGE). After protein separation, they were electrotransferred to a nitrocellulose membrane, which then was blocked with 5% non-fat dry milk in 1 × concentrated Tris–HCl saline/Tween buffer. To assess the quality of electrotransfer, the membranes were stained in Ponceau S solution and then thoroughly washed with distilled water.

After blocking, the membranes were incubated at 4 °C with anti-ADAR1 antibody (Thermo Fisher, Waltham, USA) at the dilution of 1:2500. This process was followed by washing the membranes in 1 × Tris–HCL saline/Tween buffer and placing them in a solution of secondary antibody conjugated with horseradish peroxidase (1:5000); (Santa-Cruz, California, USA). The immunochemiluminescent signal was revealed using SuperSignal West Femto Chemiluminescent Substrate (Thermo Fisher, Waltham, USA) and BioSpectrum® Imaging System 500, UVP Ltd (UVP Ltd, Upland, USA). Next, the membranes were restriped and incubated with an anti-GAPDH antibody (Santa Cruz, CA, USA) at the dilution of 1:5000, followed by incubation with a secondary goat anti-rabbit HRP-conjugated antibody (Santa Cruz, CA, USA) (dilution 1:5000). The amount of analyzed proteins was demonstrated as the investigated protein-to-GAPDH optical density ratio. Optical density was measured by using the ImageJ2x program (Rueden et al. 2017)**.**

### RNA sequencing data analysis

Data on the influence of cisplatin on ADAR1 mRNA levels in various cancer cell lines (Calu-1, A549, HeLa) was obtained from the GEO Database (Gene Expression Omnibus, https://www.ncbi.nlm.nih.gov/geo/) under the accession numbers GSE66549, GSE109214, and GSE200748. The data was analyzed using R, with the Bioconductor and GEOquery packages, and results are presented in figures and tables. Statistical analysis was conducted using the GEO2R tool available on the Gene Expression Omnibus website. The adjusted *p*-value was calculated using the Benjamini and Hochberg test, with significance set at *p* < 0.05.

### Statistical analysis

The normality of the observed data distribution was assessed by using the Shapiro–Wilk test. T-Student or ANOVA tests were used to compare the mean values of ADAR1 transcript. *p*-value < 0.05 was considered statistically significant. All statistical analyses were performed by using GraphPad Prism 8.4.3.

## Results

### ADAR1 total expression varies among different cancer cell lines

**Fig. 1:**
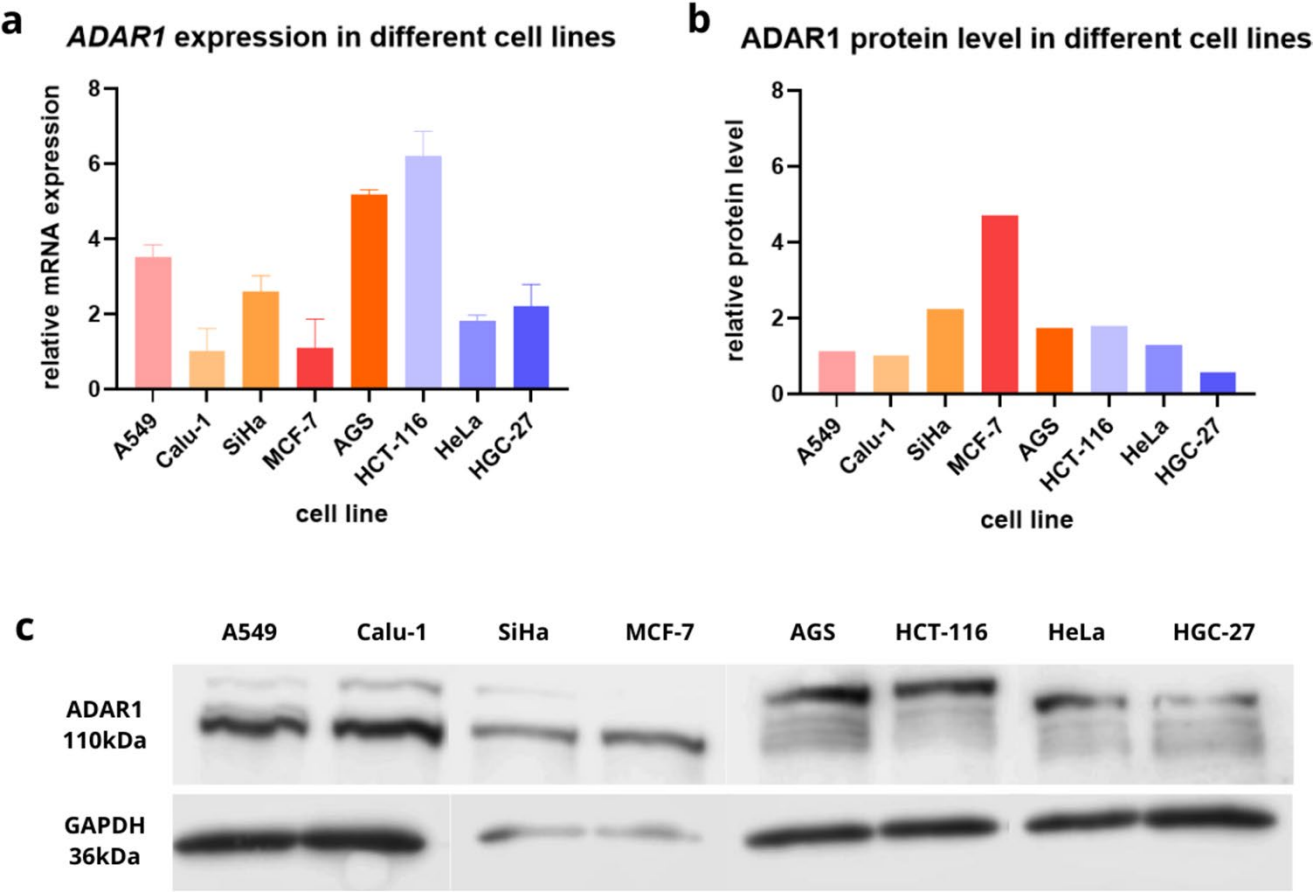
*ADAR1* expression and protein level vary in different cell lines. **a** *ADAR1* gene expression levels differ among the selected lines: A549, Calu-1, SiHa, MCF-7, AGS, HCT116, HeLa, and HGC-27. After incubation in normal conditions, total RNA from the cells was harvested and then used for gene expression assessment with RT-qPCR. Each gene transcript level is displayed as the mean multiplicity of the respective control samples ± SD. The highest mRNA levels were recorded in the colon cancer line. **b** Graph of relative ADAR1 protein level in analyzed cancer cell lines grown under normal conditions. The ADAR1 protein level differed among cell lines, of which the highest was recorded in breast cancer line MCF7. **c** Western blot analysis of ADAR1 protein expression in tumor cell lines. Each bar represents the optical density ratio of ADAR1 to GAPDH

In the first stage of our research, we assayed ADAR1 expression in different cancer cell lines, including A-549, Calu-1, SiHa, MCF-7, HeLa, HCT-116, AGS, and HGC-27. After RT-qPCR analysis, we reported significant differences in ADAR1 expression among cell lines. The highest expression was demonstrated in HCT-116 and it was 6.21 times higher compared to Calu-1, which has the lowest level of ADAR1. It was 3.53 times higher expressed in A549; 2.6 times higher in SiHa; 5.17 times higher in AGS; in Hela and HGC-27 accordingly 1.81 and 2.21 times higher than in Calu-1 (Fig. 1a, Table 1). ADAR1 expression in MCF-7 was close to Calu-1 and totaled 1.1. Statistical analysis shows significance in ANOVA test; *p* value < 0.0001. Table 2 presents results of multiple comparisons between several cell lines. HCT-116 and AGS proved to be the most significantly changed.

**Table 1:** ADAR1 relative normalized expression level in different cell lines cultured in standard conditions (37 °C, 5% CO_2_); *p*-value < 0.05 was considered statistically significant

Gene	Relative normalized expression								
	A549 (± SD)	Calu-1 (± SD)	SiHa (± SD)	MCF7 (± SD)	AGS (± SD)	HCT 116 (± SD)	HeLa (± SD)	HGC-27 (± SD)	ANOVA *p*-value
*ADAR1*	3.530 3.530	1	2.605	1.000	5.171	6.213	1.818	2.215	** < 0.0001** ****
	(± 0.316)	(± 0.614)	(± 0.413)	(± 0.769)	(± 0.141)	(± 0.655)	(± 0.150)	(± 0.574)

**Table 2:**
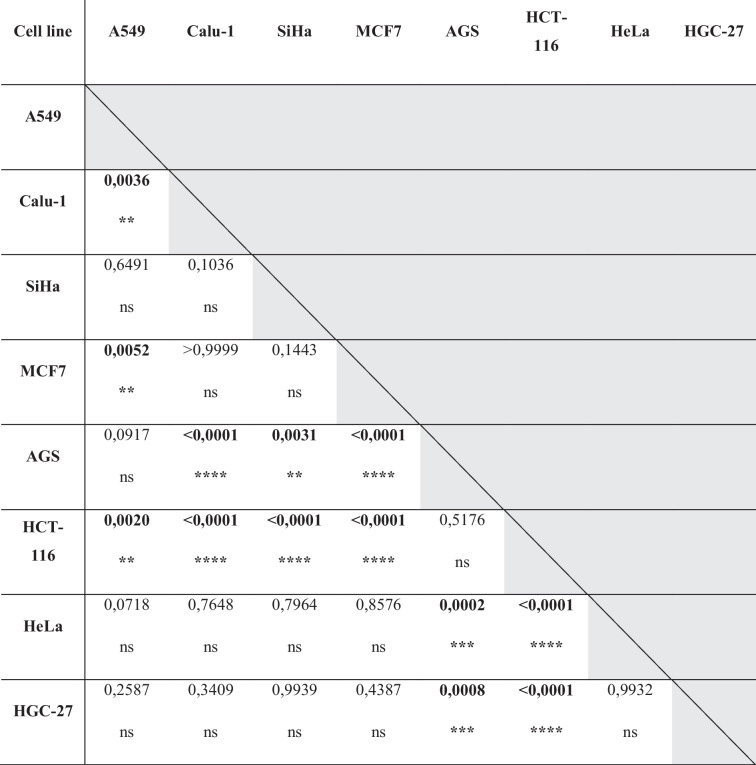
Statistical analysis of ADAR1 expression level in different cell lines cultured in standard conditions (37 °C, 5% CO_2_); *p*-value < 0.05 was considered statistically significant. Asterisk marked results significantly differ compared to each other: sample measurements (ns, not significant; *p-value < 0.05; **p-value < 0.01; ***p-value < 0.001)

Next, we measured ADAR1 protein level with western blot (WB) (Fig. 1b, c), considering the same lines as in the RT-qPCR. The highest ADAR1 protein level was in MCF-7, it was 4.71 times higher according to Calu-1, which presents one of the two lowest protein levels. A549 showed 1.12 higher score than Calu-1, SiHa protein level was 2.23 times more, then in AGS, HCT-116, and HeLa respectively 1.72, 1.79, and 1.29 times higher. Level of examined protein in HGC-27 was lower than result in Calu-1 and was equal to 0. Our results show that of ADAR1 expression on mRNA level does not correlate with protein level.

### ADAR1 expression without significant changes under the heat shock

**Fig. 2:**
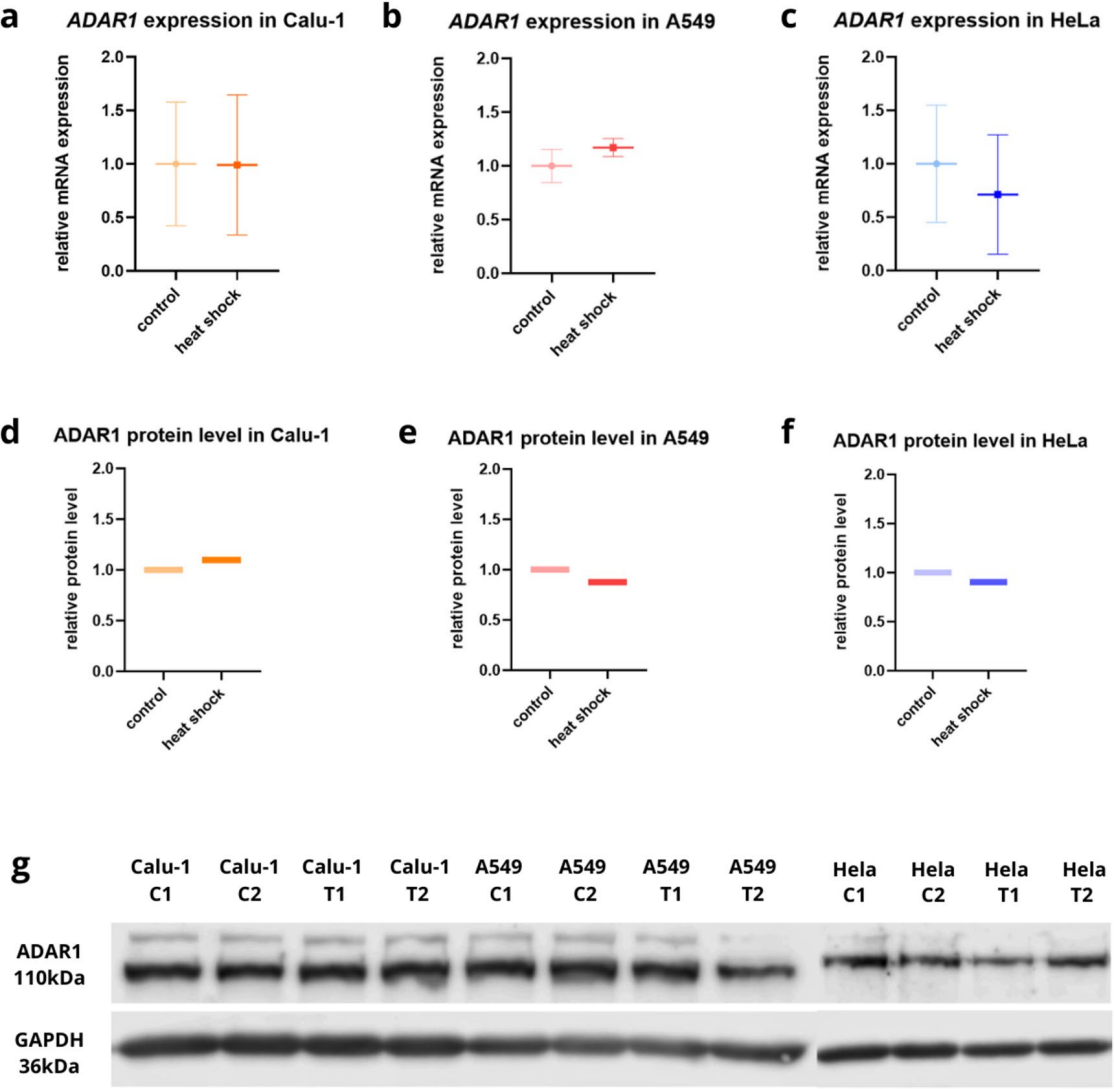
*ADAR1* expression and protein level did not change after heat shock. Relative expression of *ADAR1* in A549, Calu-1, and HeLa cells after 2 h heat shock followed by 48 h of incubation time under normal conditions. Cells were used for total RNA isolation and RT-qPCR analysis. Each gene transcript level is displayed as the mean multiplicity of the respective control samples ± SD. Heat shock did not induce significant changes in the *ADAR1* mRNA level in the A549 (**a**), Calu-1 (**b**), or HeLa cells (**c**). The relative protein level also remained constant in the aforementioned cell lines under stress conditions (**d–f**). **g** Image of western blot analysis showing that the relative protein level of ADAR1 also remained constant in Calu-1, A549, and HeLa cell lines under normal conditions (C1, C2) and under the heat shock (T1, T2)

To evaluate the effect of ADAR1 role in response to cellular stress factor, the heat shock was chosen. We selected three lines A549, Calu-1, and HeLa due to their ease of culture growth and repetitive results. RT-qPCR analysis showed no significant differences in *ADAR1* expression between cells cultured in standard conditions and under the heat shock (Fig. 2a–c, Table 3). Furthermore, there is no clear tendency of increase or downturn of *ADAR1* level between the cell lines. Protein changes assayed by WB (Fig. 2d–g) were also statistically insignificant. Noteworthy, the trend in WB analysis did not correlate with RT-qPCR results in A549 and Calu-1. In A549, ADAR1 transcript level was higher after the heat shock, but lower on the protein level. However, outcomes in Calu-1 were opposite.

**Table 3:** *ADAR1* relative normalized expression level in different cell lines cultured in standard conditions (37 °C, 5% CO_2_) and under the heat shock (43 °C for 2 h, 5% CO_2_); *p*-value < 0.05 was considered statistically significant

Gene	Conditions	Relative normalized expression		
		A549 (± SD)	Calu-1 (± SD)	HeLa (± SD)
*ADAR1*	Standard	1	1	1
		(± 0.154)	(± 0.577)	(± 0.548)
	Heat shock	1.71	0.990	0.712
		(± 0.084)	(± 0.655)	(± 0.559)
*T*-test *p*-value		*p* > 0.05	*p* > 0.05	*p* > 0.05

### ADAR1 expression without significant changes following cisplatin exposure

**Fig. 3:**
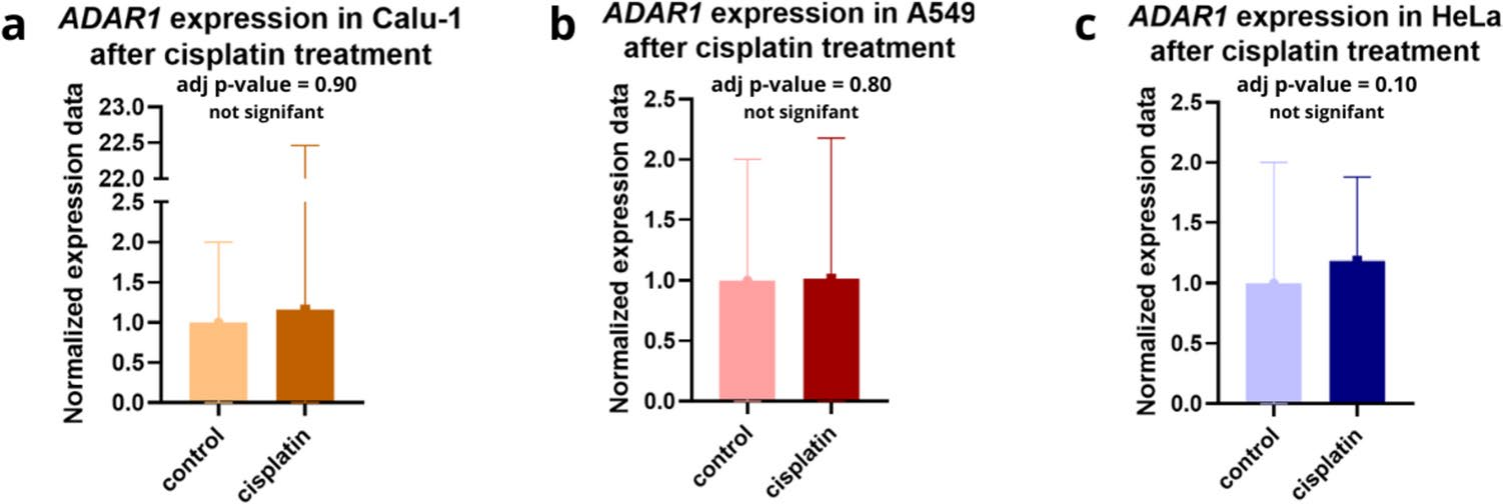
*ADAR 1* expression level did not change after cisplatin treatment. Figure present normalized expression data of *ADAR1* in Calu-1 (**a**), A549 (**b**), and HeLa 1 (**c**) cell lines after cisplatin treatment (at concentrations 10 µM, 11 µM, and 10 µM, respectively). Datasets were obtained from GEO database (Gene Omnibus, https://www.ncbi.nlm.nih.gov/geo/) under the accession numbers GSE66549, GSE109214, and GSE200748

With the aim to evaluate *ADAR1* expression levels following cisplatin treatment in the previously studied cell lines (A549, Calu-1, and HeLa), we conducted an analysis using RNA sequencing data from the Gene Expression Omnibus database (https://www.ncbi.nlm.nih.gov/geo/). The findings showed no significant changes in *ADAR1* expression levels before and after cisplatin treatment across all examined cell lines (Fig. 3c, Table 4). Our results indicate that ADAR1 mRNA and protein level are relatively stable and does not change under analyzed stress factors in examined cell lines.

**Table 4:** Statistical analysis of *ADAR1* normalized expression data in different cell lines (A549, Calu-1, and HeLa) under the treatment with cisplatin (at concentrations 11 µM, 10 µM, and 10 µM, respectively); adjusted *p*-value < 0.05 was considered statistically significant. LogFC (logarithmic Fold Change) value indicates the difference of gene expression between controls and sample groups. Statistical analysis was performed by GEO2R tool

Cell line	Adjusted *p* value	*p* value	LogFC
A549	0.798229	0.326	− 0.24597223
Calu-1	0.9014	0.49391164	− 0.170214
HeLa	0.1018469	0.0341	− 0.2493396

## Discussion

Cancer is the second leading cause of death worldwide, with almost 20 million cases recorded by the WHO in 2020 (Sung et al. 2021). Its development occurs due to mutations or genetic predisposition, as well as external factors like infection or environmental pollution. The ADAR family proteins, particularly ADAR1, may play a crucial role in the neoplastic process. High *ADAR1* expression has been linked to patient survival in several cancer types (Kung et al. 2021; Ramírez-Moya et al. 2020). However, the literature is inconsistent, whether the mRNA (Kung et al. 2021) or the protein level (Tassinari et al. 2021) indicates these predictions, which set the ground for our research. Addressing this problem, we evaluated ADAR1 expression in different cancer cell lines and found that it varies among them at both mRNA and protein levels. Importantly, we observed that the high mRNA level did not always correspond to a high protein level. Breast cancer line MCF-7 showed a relatively lower mRNA level than colon cancer HCT116, while on the protein level, ADAR1 was expressed more abundantly in the former. Our research provides valuable insight that points out in which cancer types ADAR1 may play an important role. We measured ADAR1 protein and mRNA level only in chosen commercially available lines, which is a clear limitation of our study. Although, the results are consistent with the literature suggesting breast cancer as the one with high ADAR1 expression (Kung et al. 2021) and a potential field for future research. However, additional studies that would include more cell lines or patient-derived material are necessary to confirm the role of ADAR1 in this and other types of cancers. Since high ADAR1 expression is associated with poorer survival prognoses (Kung et al. 2021; Ramírez-Moya et al. 2020), it is crucial to investigate the ADAR1 prevalence in different cancers to determine which tumors express it more intensively.

ADAR1 can influence cancer biology in three ways: by performing RNA edition (Maas et al. 2006), impacting the IFN pathways (Yang et al. 2014), and by involvement in response to cellular stress factors (Sakurai et al. 2017). All of these functions can affect gene expression, either by increasing the activity of oncogenes or decreasing the expression of tumor suppressor genes (Xu and Öhman 2018). Our research was inspired by the work of Sakurai et al.’s paper exploring the third activity—the response to stress factors. Under stress conditions, like UV radiation or heat shock, ADAR1 p110 isoform changes its cellular localization from the nucleus to the cytoplasm through MMK6-p38-MSK MAP kinases phosphorylation which regulates the apoptosis of stressed cells (Wada and Penninger 2004; Zarubin and Han 2005).

In the cytoplasm, ADAR1p110 presents a whole new activity, which is to inhibit stress-induced apoptosis not by A-to-I editing but rather by interfering in mRNA degradation. By binding to the 3′UTR of mRNA, ADAR1p110 inhibits mRNA degradation mediated by Staufen-1 protein (Sakurai et al. 2017). This results in a change in the expression of multiple genes involved in cell cycle regulation, DNA repair, or apoptosis, which prevents cells from dying. In our experiment, we aimed to fill a gap in the study by assessing whether the ADAR1 expression changes during cellular stress factors.

After subjecting cells to heat shock, we found no statistically significant differences in *ADAR1* expression levels. Similarly, cisplatin-induced cellular stress did not affect ADAR1 expression, even though there is a known correlation between increased tumor aggressiveness and chemoresistance (Wong et al. 2023; Liu et al. 2024). This lack of change in expression may be attributed to the fact that ADAR1’s functionality is influenced not only by its expression levels but also by its subcellular distribution. Our results complement Sakurai’s study (Sakurai et al. 2017), proving that the ADAR1 level remains stable and unaffected by stress-induced conditions, such as heat shock or cytostatics. It is more likely that the cellular localization, rather than the change in expression, determines ADAR1’s ability to prevent apoptosis. This finding has potential implications for cancer treatment approaches that generate stress conditions in cells like radio, laser, or microwave ablation, suggesting that targeting ADAR1 may enhance treatment efficacy (Iancu et al. 2022). Our findings suggest that future investigations could concentrate on evaluating the subcellular localization of ADAR1 and quantifying A-to-I modifications in transcripts. Another future direction for research would be exploring the impact of other stress conditions, such as radiation or another cytotoxic drugs, on ADAR1 cellular behavior and location to better understand the role of ADAR1 in cancer and in therapy.

## Conclusion

As presented, ADAR1 due to its multiple known functions is a highly compelling protein, especially as a potential marker for both cancer diagnosis and survival prediction as well as a target for future therapies. Our study demonstrated different expression of ADAR1 on mRNA and protein levels among chosen cell lines, indicating which types of cancer may be a promising target for novel therapeutic approaches. Protein and mRNA levels remained unchanged in the selected cell lines after the heat shock, suggesting that the expression of *ADAR1* is independent from this stress factor. In the same cell lines, cisplatin, an anticancer medication, had no effect on the expression of ADAR1. It would be beneficial to demonstrate similar stability during chemotherapy with other cytostatics or radiotherapy to further increase the evidence base for such speculations. Although examined cultures represent just a tiny percentage of the vast field of oncology, our results lay the groundwork for future research on the function of ADAR1 in respective tumors with a novel approach.
